# Acceptability and utilization of family planning benefits cards by youth in slums in Kampala, Uganda

**DOI:** 10.1186/s40834-019-0092-2

**Published:** 2019-08-05

**Authors:** Afra Nuwasiima, Elly Nuwamanya, Janet U. Babigumira, Robinah Nalwanga, Francis T. Asiimwe, Joseph B. Babigumira

**Affiliations:** 1 GHE Consulting, P.O Box 27011, Kampala, Uganda; 2 Department of Global Health, University of Washington, 1959 NE Pacific Street, Health Sciences Building F-151-B, Box 357630, Seattle, WA 98195 USA

**Keywords:** Family planning, Contraception, Acceptability, Utilization, Benefits cards, Slums, Youth, Discontinuation

## Abstract

**Background:**

This study was conducted to test the acceptability and utilization of family planning benefits cards (FPBCs) as incentives to increase family planning uptake among youth living in urban slums in Uganda.

**Methods:**

We conducted a one-year pilot study with two sub-studies on acceptability and utilization of FPBCs. The acceptability study utilized a quantitative cross-sectional design and was part of a baseline household survey while the utilization study was a primary analysis of claims and clinic data. We performed descriptive analyses and analyses of the association between different variables using binary logistic regression.

**Results:**

The acceptability study included 280 eligible females. The majority were married (52%), Christian (87%), and aged 20 and above (84%). Acceptability of the program was high (93%). Seventy-two percent of females used the card at least once to access reproductive health services. Twenty-seven percent of female users discontinued family planning and 14% changed family planning methods during the study. Female users of short-term contraceptive methods were 11 times more likely to discontinue use of FPBCs compared to those who used long-term methods (adjusted OR = 10.9, *P* = 0.011). Participants in professional/managerial employment were 30 times more likely to discontinue compared to the unemployed (adjusted OR = 30.3, *P* = 0.015). Participants of parity equal to two were 89% less likely to discontinue use of FPBCs compared to those of parity equal to zero (adjusted OR = 0.1, *P* = 0.019).

**Conclusion:**

Family planning benefits cards, deployed as incentives to increase uptake of family planning, exhibited high acceptability and utilization by youth in urban slums in Uganda. There was evidence that use of short-term contraception methods, professional employment, and lower parity were associated with discontinuation of modern family planning methods after initial enrolment.

**Trial registration:**

MUREC1/7 No. 10/05–17. Registered 19th, July 2017.

## Background

Thirty-five percent of Ugandan women aged 15 to 49 currently use modern contraception and 28% have an unmet need for contraception [1]. The use of modern contraception is higher among the richest quintile of women (49%) and among urban women (41%) compared to the poorest quintile of women (22%) and women living in rural areas (33%) [1]. Uganda has a total fertility rate of 5.4, one of the highest in the world, and 43% of women have unintended pregnancies [1, 2]. Most unintended pregnancies are due to lack of contraceptives (88%) as compared to contraceptive failure (12%) [3].

The high level of unmet need is accelerated by population growth, shortages in family planning services, inadequate family planning counselling, and lack of youth-friendly family planning services [4–6]. Despite the high knowledge and awareness of modern contraceptive methods (90%), utilization remains low due to low levels of education, lack of knowledge of the side effects of different contraceptive methods, and prohibitive cultural, social and religious norms [5, 7, 8].

The Government of Uganda has pledged to increase uptake of modern contraception to 50% and reduce the unmet need to 10% by increasing access to family planning information, targeting youth, and addressing the social and cultural misconceptions about contraception [2]. With support from the World Health Organization (WHO), the government is implementing youth friendly corners—designated spots for youth support—at health facilities to increase uptake of sexual and reproductive health services, including contraception [6]. The early results from this program suggest an increase in the proportion of youth with access to contraception, especially among informal workers such as waitresses and hair dressers [6, 9, 10].

The use of benefits as a vehicle for healthcare access is not novel. Non-cash strategies such as redeemable vouchers have been found to increase uptake of family planning and maternal health services [11–13]. Several other social franchising strategies that incentivize the use of contraceptives are fundamental to helping low- and middle-income countries (LMICs) achieve global family planning targets [14–16]. The objective of this study was to assess the acceptability and utilization of family planning benefits cards as a vehicle for increasing contraceptive coverage in the setting of urban slums in Kampala, Uganda.

## Methods

### Study setting

The study was conducted in Kifumbira slum in Kampala, Uganda’s capital city, from September 08, 2017 to March 07, 2018. We purposively selected Kifumbira slum as the intervention area because most of its residents fall in the lowest wealth quintile and record high levels of unmet need for contraception [17]. A significant proportion of the slum population is unemployed and thus unable to afford primary medical care through out of pocket expenditure [18].

### Intervention description

The study was conducted as part of an impact evaluation of a family planning benefits cards (FPBC) program, a partnership between: (1) GHE Consulting, a research firm; (2) International Medical Link (IML), a health insurance firm; (3) community clinics and pharmacies; and (4) community health workers (CHWs). A description of the FPBC program and an evaluation protocol have been described in detail in a previous publication [19]. GHE Consulting was the project coordinator and the principal for study design, data collection, and data analysis. GHE Consulting engaged with different public and private stakeholders including the Uganda Ministry of Health, district and local officials, corporate firms, and donor agencies.

IML designed and managed the FPBC system and was responsible for conducting quality assurance, establishing partnerships with community health centers and pharmacies, managing and paying claims for program services, and providing data to GHE Consulting. The CHWs were responsible for mobilizing and sensitizing community members about the FPBC program, performing family planning counselling, and emphasizing the importance of using family planning. The CHWs received refresher training on comprehensive family planning services at months one and three of the project.

Through the partnership with IML and the partner clinics and pharmacies, the FPBC program provided family planning services to the youth aged 18 to 30 years. Participants received a FPBC and a list of health facilities and pharmacies where it could be used. The FPBC contained the beneficiary’s photograph, names, and a card number. The FPBC granted beneficiaries access to counselling and guidance, non-permanent contraceptive methods, pregnancy testing, and HIV testing and counselling. The FPBC provided the covered services free of charge for a period of six months.

### Study design

The study was a one-year pilot with two sub-studies: the acceptability study and the utilization study. The acceptability study utilized a quantitative cross-sectional design and was part of a baseline household survey of contraceptive use among youth in the target areas. Baseline survey participants were assessed for eligibility to participate in the FPBC program. Eligible participants were: 1) aged between 18 to 30 years, 2) non-users of modern contraceptive methods, 3) sexually active and not currently pregnant, and 4) willing to provide informed consent. The utilization study used claims and clinic data obtained from FPBC users.

### Sampling and sample size

We used convenience sampling to recruit participants for the household survey with a target of including 200 to 300 individuals as recipients and beneficiaries of the FPBC. This number was based on projections related to the available resources for the project. The sample size for the utilization study was determined by the number of FPBC beneficiaries.

### Measurement of study outcomes

Acceptability was measured by estimating the proportion of eligible participants who accepted the FPBC. Individuals that refused the FPBC were probed further to identify the reasons for refusal. Categories of reasons for refusal were created for the analysis.

Utilization of the FPBC was measured by the number of beneficiaries that used the card for at least one of the program services in six months. We reviewed the participant utilization data to assess the proportion of participants that changed contraceptive methods and/or those that discontinued the use of contraceptive methods in the six months period. Participants were asked about the reasons for change or discontinuation of contraceptive methods.

### Community health worker recruitment and training

The study recruited and trained ten CHWs on comprehensive family planning services at baseline, with a refresher training at three months, to perform community mobilization and sensitization about family planning and the FPBC program.

### Community mobilization and sensitization

CHWs continuously patrolled their assigned zones within the intervention area, conducting door-to-door sensitization about the FPBC program. We also conducted a radio campaign at the start of the program to mobilize the community to participate in the program.

### Data collection and survey instruments

Separate instruments were utilized for the acceptability and utilization studies. The acceptability study instrument contained questions on socio-economic and demographic characteristics, willingness to join the FPBC program and reasons for refusal to join the program for those that declined. Data were collected by research assistants who were recruited and trained on the survey tools, family planning, and the ethical conduct of research including human subjects. Data were collected using open data toolkit (ODK) installed on android smart phones.

Utilization data were collected using medical records designed by IML, the insurance provider. The forms collected participant’s card numbers, names, purpose of facility visit, family planning method utilized, and other services rendered. Additionally, each participant was followed up by either phone call or in-person visit to verify the data obtained from medical records. During verification calls or visits, reasons for discontinuation, change of family planning method, and non-use of the FPBC were probed.

### Data analysis

The analysis was performed using Microsoft Excel and STATA version 13.0 (Stata Corporation, College Station, Texas, USA). We performed descriptive analyses of demographic characteristics using means and proportions. Bivariate analyses using the chi-square test of association were performed to further characterize the study sample by acceptability and utilization of FPBCs.

We assessed the association between different variables and the two outcome variables i.e. acceptability and utilization of the FPBCs using univariate and multivariate logistic regression (outcome variables coded as 0/1). Both adjusted and unadjusted odds ratios are reported with their corresponding *p*-values and confidence intervals (CI). All the study results were considered statistically significant at the 5% level.

### Ethics statement

**Fig. 1:**
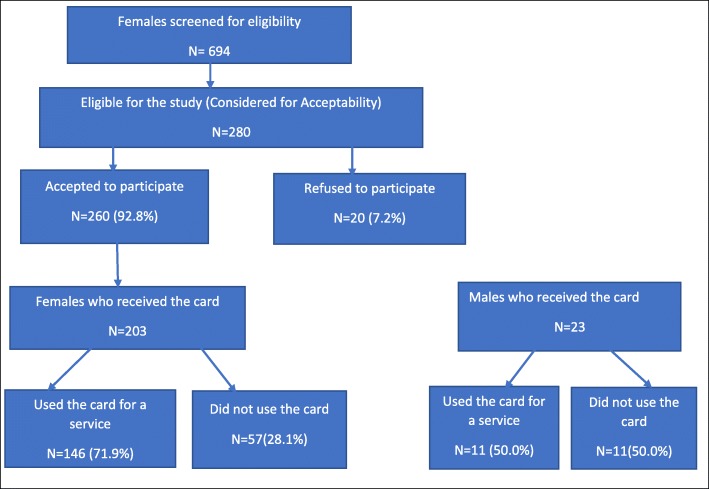
Study participant flow diagram showing both acceptability and utilization

The study was approved by the Mbarara University of Science and Technology (MUST) ethics review committee and the Uganda National Council of Science and Technology (UNCST). The study also received regulatory clearances from the Uganda Ministry of Health and local authorities. All study participants provided informed consent. All personal identifiers such as names and, photos were stored separately from the survey data and were password protected (Fig. 1).

## Results

### Participant characteristics

Table 1 shows the demographic characteristics of study participants by acceptance of the FPBC (all participants, participants that accepted the FPBC, and participants that refused the FPBC). Most participants (48%) were aged above 24 years, married (52%), Christian (87%), and had attained a secondary level of education (50%). Participants were predominantly unemployed (44%) or had a professional job (26%). Most participants’ partners had attained at least a secondary level of education (76%) and were employed as salesmen or traders (39%). The distribution of parity was para one (28%), para zero (26%) and para two (20%).

**Table 1:** Demographic characteristics of participants in a household survey of potential family planning benefits cards beneficiaries showing all participants and participants by acceptability status (accepted vs. refused)

Characteristic	Over all *N* = 280	Accepted *N* = 260	Refused *N* = 20	*P* value*
Age group, *n* (%)				
< 20	45 (16.07)	42 (16.15)	3 (15.00)	0.668
20–24	100 (35.71)	91 (35.00	9 (45.00)	
> 24	135 (48.21)	127 (48.85)	8 (40.00)	
Marital status, *n* (%)				
Married	145 (51.79)	140 (53.85)	5 (25.00)	0.023
Separated/Divorced	39 (13.93)	37 (14.23)	2 (10.00)	
Widow	1 (0.36)	1 (0.38)	0 (0.00)	
Never Married	95 (33.93)	82 (31.54)	13 (65.00)	
Religion, *n* (%)				
Christian	244 (87.14)	228 (87.69)	16 (80.00)	0.057
Muslim	35 (12.50)	32 (12.31)	3 (12.31)	
Others	1 (0.36)	0 (0.00)	1 (5.00)	
Education level, *n* (%)				
No education	22 (7.86)	19 (7.31)	3 (15.00)	0.061
Primary	79 (28.21)	76 (29.23)	3 (15.00)	
Secondary	140 (50.00)	132 (50.77)	8 (40.00)	
More than Secondary	39 (13.93)	33 (12.69)	6 (30.00)	
Partner’s Education level, *n* (%)				
No education	6 (4.14)	6 (4.29)	0 (0.00)	0.112
Primary	17 (11.72)	15 (10.71)	2 (40.00)	
Secondary	82 (56.55)	81 (57.86)	1 (20.00)	
More than Secondary	28 (19.31)	27 (19.29)	1 (20.00)	
Don’t know	12 (8.28)	11 (7.86)	1 (20.00)	
Occupation, *n* (%)				
Unemployed	122 (43.57)	109 (41.92)	13 (65.00)	0.004
Farming	1 (0.36)	0 (0.00)	1 (5.00)	
Trading	62 (22.14)	62 (23.85)	0 (0.00)	
Professional	73 (26.07)	68 (26.15)	5 (25.00)	
Other jobs	22 (7.9)	21 (8.08)	1 (5.00)	
Partner’s Occupation, *n* (%)				
Unemployed	6 (4.1)	6 (4.29)	0 (0.00)	0.009
Farming	1 (0.7)	0 (0.00)	1 (20.00)	
Sales/Trading	56 (38.6)	54 (38.57)	2 (40.00)	
Professional	45 (31.0)	45 (32.14)	0 (0.00)	
Other jobs	33 (22.8)	32 (22.86)	1 (20.00)	
Don’t know	4 (2.8)	3 (2.14)	1 (20.00)	
Parity, *n* (%)				
0	74 (26.4)	64 (24.62)	10 (50.00)	0.114
1	78 (27.9)	72 (27.69)	6 (30.00)	
2	57 (20.4)	55 (21.15)	2 (10.00)	
3	43 (15.4)	41 (15.77)	2 (10.00)	
4 and above	28 (10.0)	28 (10.77)	0 (0.00)	

*p-value of difference in demographics comparing participants who accepted vs. participants who refused family planning benefits cards

### Acceptability of the FPBC program

**Fig. 2:**
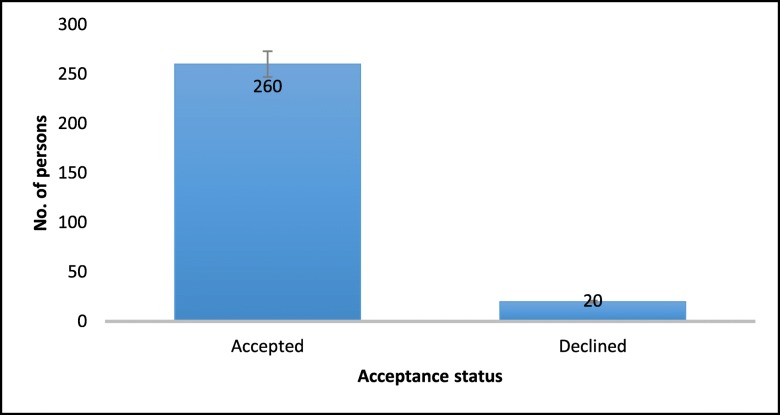
Number of persons by acceptability status

Acceptability results are shown in Fig. 2. A larger proportion (93%) of the women included in the study accepted participation in FPBC program. Table 1 shows the results of the chi-square test of association between acceptability and demographic characteristics. The results shown that acceptability of the FPBC program was higher among married women compared to never married women (54% vs. 32%, *P* = 0.023). The unemployed were more likely to refuse the FPBC program than professionals were (65% vs. 25%, *P* = 0.004). Demographic characteristics such as age group, religion, education level, and parity were not significantly associated with acceptability.

**Fig. 3:**
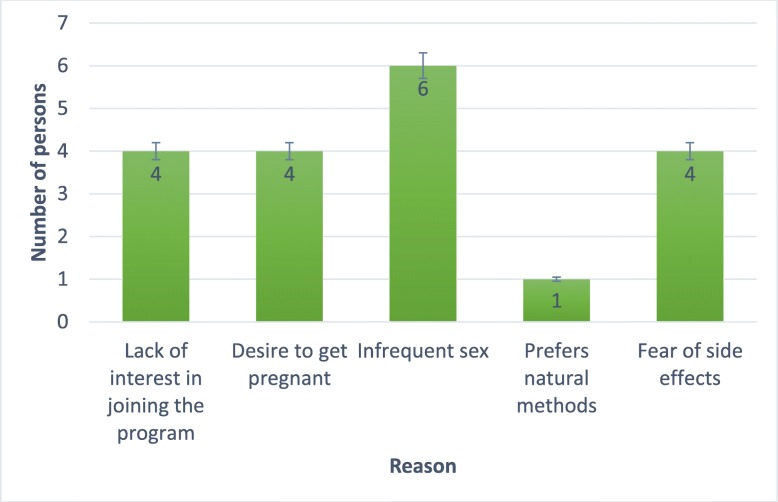
Reasons for declining to join the FPBC program

Figure 3 shows the different reasons for declining participation in the FPBC program. The results show that infrequent sex, (*n* = 6 (30%)), lack of interest in joining the program, (*n* = 4 (20%)), desire to get pregnant, (*n* = 4 (20%)) and fear of side effects of contraceptive use, (*n* = 4 (20%)) were the reasons for declining to join the FPBC program. Findings from the logistic regression showed that none of the demographic characteristics were significantly associated with acceptability of the FPBC at the univariate level.

### Utilization of the FPBC

Table 2 shows the distribution of FPBC use among recipients stratified by gender for the different demographic characteristics. Overall, 72% of females and 50% of males used the card to access at least one service at the partner clinics in the six months program period. Among the females, most of the card users were aged 25 years (47%), married (63%), and had attained a secondary level of education (64%). Among the males, most of the card users were also aged 25 years and above (45%), married (55%), and had attained a secondary level of education (64%).

**Table 2:** Utilization of family planning benefits cards among participants who accepted the cards by select demographic characteristics

Characteristic	Females		Males	
	Over all *N* = 203	Used, *N* = 146 (71.9%)	Overall, *N* = 22	Used, *N* = 11 (50.0%)
Age group, *n* (%)				
< 20	26(12.8)	20(13.7)	4(18.2)	3(27.3)
20–24	81(39.9)	57(39.0)	8(36.4)	3(27.3)
> 24	96(47.3)	69(47.3)	10(45.4)	5(45.4)
Marital status, *n* (%)				
Married	129(63.5)	92(63.0)	10(45.4)	6(54.5)
Separated/Divorced	22(10.8)	20(13.7)	4(18.2)	1(9.1)
Widow	1(0.5)	1(0.7)	–	–
Never Married	51(25.1)	33(22.6)	8(36.4)	4(36.4)
Education level, *n* (%)				
No education	9(4.4)	4(2.7)	2(9.1)	0(0.0)
Primary	55(27.1)	36(24.6)	5(22.7)	2(18.2)
Secondary	118(58.1)	93(63.7)	12(54.5)	7(63.6)
More than Secondary	21(10.3)	13(8.9)	3(13.6)	2(18.2)

Table 3 presents the reasons for none use of the FPBC in the six months study period for the female participants who were available for interview. Desire to get pregnant (35%) and infrequent sex (25%) were the main reasons for non-use of the FPBC.

**Table 3:** Reasons for non-use of family planning benefits cards

Reason	Distribution, *n* (%)
*Desire for next child/Got pregnant before use*	*7(35.0)*
*No frequent sex/Abstaining*	*5(25.0)*
*Card was misplaced before use*	*3(15.0)*
*Fear of side effects*	*2(10.0)*
*Received the card when already started on a new family planning method*	*2(10.0)*
*Was not attended to at first visit and did not go back*	*1(5.0)*

### Association between utilization and demographic characteristics

We fit a binary logistic regression model using card utilization (used = 1 vs. not used = 0) as the outcome variable and demographic characteristics as covariates. The univariate logistic regression results showed that women with secondary education were 5 times more likely to use the FPBC compared to those of no education (OR = 4.65, *P* = 0.030). The rest of the covariates were not significantly associated with utilization of the FPBCs. None of the demographic characteristics were significantly associated with the card utilization at multivariate regression model. The results of this analysis are presented in Table 4.

**Table 4:** Logistic regression analysis of the association of utilization of the FPBC with the demographic characteristics

Characteristic	Utilization of the family planning benefits card			
	Unadjusted OR (*P* value)	95% CI	Adjusted OR (*P* value)	95% CI
Age; Ref = < 20				
20–24	0.713(0.519)	(0.254, 1.995)	0.667(0.533)	(0.186, 2.386)
> 24	0.767(0.605)	(0.278, 2.115)	0.803(0.771)	(0.183, 3.530)
Marital status; Ref = Married				
Separated/Divorced	4.022(0.070)	(0.895, 18.075)	2.116(0.566)	(0.164, 27.299)
Never Married	0.737(0.386)	(0.370, 1.469)	0.323(0.324)	(0.034, 3.043)
Religion; Ref = Christian				
Muslim	0.789(0.574)	(0.346, 1.800)	0.759(0.562)	(0.300, 1.922)
Education level; Ref = No education				
Primary	2.368(0.236)	(0.568, 9.871)	1.342(0.726)	(0.259,6.956)
Secondary	4.65(0.030)*	(1.162, 18.612)	3.154(0.153)	(0.653, 15.232)
More than secondary	2.031(0.380)	(0.417, 9.886)	2.315(0.366)	(0.375, 14.301)
Partner’s Education level; Ref = No education				
Primary	1.000(1.000)	(0.984, 10.166)	1.155(0.918)	(0.075, 17.770)
Secondary	1.033(0.970)	(0.188, 5.691)	1.202(0.869)	(0.135, 10.681)
More than secondary	0.733(0.751)	(0.108, 4.992)	0.815(0.870)	(0.071, 9.373)
Don’t know/Not married	1.145(0.876)	(0.207, 6.334)	3.558(0.352)	(0.245, 51.590)
Occupation; Ref = Unemployed				
Sales/Trading	1.553(0.279)	(0.700, 3.446)	1.524(0.349)	(0.631, 3.678)
Professional/Managerial	0.769(0.499)	(0.360, 1.644)	0.674(0.384)	(0.277, 1.639)
Other jobs	0.694(0.588)	(0.185, 2.598)	0.716(0.677)	(0.148, 3.453)
Partner’s Occupation level; Ref = Unemployed				
Farming	0.625(0.683)	(0.065,5.980)	0.335(0.431)	(0.022, 5.084)
Sales/Trading	0.781(0.836)	(0.076,8.041)	0.512(0.631)	(0.033, 7.822)
Professional/Managerial	0.603(0.658)	(0.065, 5.632)	0.242(0.333)	(0.014, 4.282)
Other jobs	0.500(0.676)	(0.019, 12.898)	0.241(0.473)	(0.005, 11.718)
Don’t know/not married				
Parity; Ref = 0				
1	1.671(0.285)	(0.652, 4.284)	0.151(0.544)	(040, 5.635)
2	1.475(0.373)	(0.627, 3.472)	0.956(0.949)	(0.242, 3.778)
3	0.657(0.407)	(0.243, 1.774)	0.485(0.372)	(0.099,2.375)
4 and above	0.839(0.757)	(0.275, 2.557)	0.859(0.868)	(0.142, 5.198)

*OR* Odds Ratio | *significant at 95% confidence interval (CI)

### Change and discontinuation of family planning methods

Table 5 shows the number of participants that changed family planning method among those who used the FPBC to access family planning services. The results show that 21 (14%) female card users changed to another type of family planning method. Ten (48%) females changed from a short-term to the long-term method and eight (38%) participants changed from one short-term method to another. Two women (10%) changed from implant to injectables and one woman changed from intrauterine device (IUD) to implant.

**Table 5:** Number and of female participants that changed family planning method among users of family planning benefits cards

Changed family planning method, *N* (%) = 21 (14.4%)	Distribution, *n* (%)
Short term to long term	10 (47.6)
*Pills to Implant*	*3 (14.3)*
*Condom to Implant*	*1 (4.8)*
*Injectables to Implant*	*6 (28.5)*
Short term to short term	8 (38.1)
*Pill to Injectables*	*7 (33.3)*
*Emergency contraception to Injectables*	*1 (4.8)*
Long term to short term	2 (9.5)
*Implant to Injectables*	*2 (9.5)*
Long term to long term	1 (4.8)
*IUD to Implant*	*1 (4.8)*

Table 6 shows the probability of discontinuation of family planning by family planning method. The majority of those that discontinued (93%) discontinued from pills (47.5%), injectables (28%), emergency contraception (10%) and condoms (8%). Only three participants discontinued from a long-term family planning method (implant).

**Table 6:** Number of participants that discontinued the use of family planning among users of family planning benefits cards

Discontinuation, *N* (%) = 40	Distribution, *n* (%)
Discontinued from a short-term method	37 (92.5)
* Pills*	*19 (47.5)*
* Injectables*	*11 (27.5)*
* Condoms*	*3(7.5)*
* Emergency contraception*	*4 (10.0)*
Discontinued from a long-term method	3 (7.5)
* Implants*	*3 (7.5)*

### Association of discontinuation of family planning use by demographic characteristics and method type

We fit a binary logistic regression model with discontinuation (discontinued = 1 vs. not discontinued = 0) as the outcome variable, and the demographic characteristics plus family planning method as covariates. The types of family planning were classified as long- and short-term methods. The results are presented in Table 7. Type of family planning method, age group, marital status, education, partner’s occupation and parity were significantly associated with family planning discontinuation in univariate analyses. For example, participants above 24 years were 73% less likely to discontinue compared to those who were aged below 20 years (OR = 0.27, *P* = 0.016) and those who were single were approximately 3 times more likely to discontinue compared to those who were married (OR = 2.906, *P* = 0.015).

**Table 7:** Logistic regression analysis of the association of discontinuation of family planning method with the demographic characteristics and type of family planning method

Characteristic	Discontinuation of family planning			
	Unadjusted OR (*P* value)	95% CI	Adjusted OR (*P* value)	95% CI
Method type; Ref = Long-term				
Short-term	4.703(0.016)*	(1.342, 16.474)	10.889(0.011)*	(1.723, 68.837)
Age; Ref = < 20				
20–24	0.307(0.033)*	(0.104, 0.910)	0.520(0.412)	(0.109, 2.483)
> 24	0.272(0.016)*	(0.094, 0.784)	0.299(0.244)	(0.039, 2.281)
Marital status; Ref = Married				
Separated/Divorced	2.124(0.161)	(0.740, 6.096)	> 100(0.994)	(0, infinity)
Never Married	2.906(0.015)*	(1.227, 6.886)	> 100(0.994)	(0, infinity)
Religion; Ref = Christian				
Muslim	1.122(0.828)	(0.398, 3.157)	1.079(0.920)	(0.243, 4.786)
Education level; Ref = No education				
Primary	1.435(0.766)	(0.134, 15.417)		
Secondary	1.000(1.000)	(0.099, 10.093)	8.322(0.104)	(0.648, 106.920)
More than secondary	1.875(0.625)	(0.150, 23.396)	2.149(0.476)	(0.263, 17.588)
Partner’s Education level; Ref = No education				
Primary	1.000(1.000)	(0.079,12.557)	1 (empty)	–
Secondary	0.103(0.021)*	(0.015, 0.712)	> 100(0.994)	(0, infinity)
More than secondary	0.381(0.383)	(0.043, 3.338)	> 100(0.995)	(0, infinity)
Don’t know/Not married	0.368(0.291)	(0.057, 2.363)	> 100(0.993)	(0, infinity)
Occupation; Ref = Unemployed				
Sales/Trading	1.103(0.825)	(0.461, 2.637)	1.788(0.371)	(0.501, 6.382)
Professional/Managerial	0.784(0.642)	(0.281, 2.184)	2.350(0.291)	(0.481, 11.474)
Other jobs	3.733(0.109)	(0.747, 18.656)	30.310(0.015)*	(1.952, 470.437)
Partner’s Occupation level; Ref = Unemployed				
Sales/Trading	0.274(0.010)*	(0.102, 0.738)	2.136(0.500)	(0.236, 19.344)
Professional/Managerial	0.846(0.738)	(0.318, 2.249)	1.449(0.760)	(0.134, 15.683)
Parity; Ref = 0				
1	0.292(0.020)*	(0.103, 0.826)	0.157(0.031)*	(0.029, 0.845)
2	0.284(0.011)*	(0.108, 0.748)	0.112(0.019)*	(0.018, 0.697)
3	0.06(0.012)*	(0.007, 0.537)	0.039(0.039)*	(0.002, 0.852)
4 and above	0.159(0.031)*	(0.030, 0.844)	0.039(0.037)*	(0.002, 0.824)

*OR* Odds Ratio | *significant at 95% confidence interval (CI)

In multivariable analyses, participants who used a short-term method were 11 times more likely to discontinue compared to those who used a long-term method (adjusted OR = 10.89, *P* = 0.011). Female participants in professional/managerial employment were 30 times more likely to discontinue compared to those who were unemployed (Adjusted OR = 30.31, P = 0.015). Participants of parity equal to two were 89% less likely to discontinue compared to those of parity equal to zero (Adjusted OR = 0.11, *P* = 0.019) and participants of parity equal to one were also 84% less likely to discontinue compared to those of parity equal to zero (adjusted OR = 0.18, *P* = 0.031). Participants of parity equal to three were 96% less likely to discontinue compared to those of parity equal to zero (Adjusted OR = 0.04, *P* = 0.039).

## Discussion

Family planning benefits cards were acceptable to the majority of female youth in urban slums in Kampala, Uganda. Women who refused to join the program gave reasons such as infrequent sex, lack of interest, fear of side effects of contraception, and desire to have a child. These have been cited as reasons for discontinuation or never use of contraceptives in Uganda [20] and Ethiopia [21]. Utilization of the family planning benefits cards was high (70%), particularly more so among females (72%) than males (50%). These acceptability and utilization results provide evidence to suggest that FPBCs have the potential to create demand for family planning and other sexual and reproductive health services. This finding is consistent with the results of a prior review that found an incentives-based voucher program to lead to increased demand for sexual and reproductive health services in Uganda [13].

Most incentive-based family planning initiatives limit the choices of clients by designing method-specific family planning programs [12–14]. Our study allowed clients to make their preferred choices amongst the different non-permanent family planning tools and services available on the Ugandan market. The results suggested preference by women for more short-term methods like injectables and pills to long-term methods like IUDs and implants. This is supported by the predominance of desire to get pregnant among the reasons of non-use (35%) and a relatively young (and therefore more fertile) population in the study, which was by design. The results suggested the FPBC program in its current design was less appealing to males. Future incentives studies might explore alternative models for increasing male participation in the uptake of reproductive health programs including family planning.

The study allowed us to measure the rates of change and discontinuation of family planning methods. Approximately 1 in 10 female users of modern contraception changed methods and approximately 1 in 3 discontinued the use of family planning. Previous studies have also suggested that approximately 1 in 3 women who start a modern contraception change methods with in the first year [22, 23]. Our study results indicate that the main reasons for changing methods included discomfort and side effects while the main reasons for discontinuation included the desire to get pregnant, contraceptive failure, side effects, and infrequent sex/abstinence. Fear of side effects remains a strong barrier to both initiation and adherence to modern family planning methods as highlighted in prior studies [22, 23].

Our results suggest that the use of short-term methods, lower parity and professional employment were associated with discontinuation of modern family planning. Higher parity, in this case para 1 and 2, may be associated with lower desire to get pregnant and professional women may have felt more empowered to discontinue or change family planning, particularly short-term methods that are easier to discontinue. Although the FPBCs covered all services including removal of IUDs and implants, the additional visit to facilities may be a disincentive to discontinue, leading to higher rates of discontinuation for short-term methods. Longer, longitudinal studies are needed to better understand the timing and causes of change and discontinuation of non-permanent modern contraceptives. Such studies will complement the evidence base to inform recommendations to improve the uptake of family planning and minimize discontinuation, consistent with the priorities of the Uganda government.

The study was conducted in the setting of urban slums in Kampala among 18 to 30 year olds. Therefore, the results may not be generalizable to other groups of women or youth in the country. Although we report results on change and discontinuation of family planning methods, these data should be interpreted with caution given the short benefits period of six months. Additionally, while there is evidence of high acceptability and utilization, a cost-effectiveness analysis of the FPBCs, complete with assessment of alternative paths to sustainability of such a program, is needed.

## Conclusions

The family planning benefits cards provided to urban youth in Uganda showed high acceptability and utilization. There was evidence that use of short-term contraception methods, professional employment, and lower parity were associated with discontinuation of modern family planning methods after initial enrolment. Longer studies will better characterize the reasons for discontinuation of family planning and the potential for inclusion of a wider range of sexual and reproductive health services to increase the demand for and use of family planning benefits cards.

## Data Availability

All the data presented in this study is available upon request from the corresponding author.

## Abbreviations

CHWs: Community Health Workers
FPBC: Family Planning Benefits Card
IML: International Medical Link
IRB: Institutional Review Board
IUD: Intra-Uterine Device
LMICs: Low- and Middle-Income Countries
MUST: Mbarara University of Science and Technology
ODK: Open Data Kit
OR: Odds Ratio
UNCST: Uganda National Council of Science and Technology
WHO: World Health Organization
